# Early PTSD Symptom Trajectories: Persistence, Recovery, and Response to Treatment: Results from the Jerusalem Trauma Outreach and Prevention Study (J-TOPS)

**DOI:** 10.1371/journal.pone.0070084

**Published:** 2013-08-22

**Authors:** Isaac R. Galatzer-Levy, Yael Ankri, Sara Freedman, Yossi Israeli-Shalev, Pablo Roitman, Moran Gilad, Arieh Y. Shalev

**Affiliations:** 1 Center for Traumatic Stress Studies, Hadassah University Hospital, Jerusalem, Israel; 2 Department of Psychiatry, NYU School of Medicine, New York, New York, United States of America; 3 School of Social Work, Bar Ilan University, Ramat Gan, Israel; University of Tasmania, Australia

## Abstract

**Context:**

Uncovering heterogeneities in the progression of early PTSD symptoms can improve our understanding of the disorder's pathogenesis and prophylaxis.

**Objectives:**

To describe discrete symptom trajectories and examine their relevance for preventive interventions.

**Design:**

Latent Growth Mixture Modeling (LGMM) of data from a randomized controlled study of early treatment. LGMM identifies latent longitudinal trajectories by exploring discrete mixture distributions underlying observable data.

**Setting:**

Hadassah Hospital unselectively receives trauma survivors from Jerusalem and vicinity.

**Participants:**

Adult survivors of potentially traumatic events consecutively admitted to the hospital's emergency department (ED) were assessed ten days and one-, five-, nine- and fifteen months after ED admission. Participants with data at ten days and at least two additional assessments (n = 957) were included; 125 received cognitive behavioral therapy (CBT) between one and nine months.

**Approach:**

We used LGMM to identify latent parameters of symptom progression and tested the effect of CBT on these parameters. CBT consisted of 12 weekly sessions of either cognitive therapy (n = 41) or prolonged exposure (PE, n = 49), starting 29.8±5.7 days after ED admission, or delayed PE (n = 35) starting at 151.8±42.4 days. CBT effectively reduced PTSD symptoms in the entire sample.

**Main Outcome Measure:**

Latent trajectories of PTSD symptoms; effects of CBT on these trajectories.

**Results:**

Three trajectories were identified: *Rapid Remitting* (rapid decrease in symptoms from 1- to 5-months; 56% of the sample), *Slow Remitting* (progressive decrease in symptoms over 15 months; 27%) and *Non-Remitting* (persistently elevated symptoms; 17%). CBT accelerated the recovery of the Slow Remitting class but did not affect the other classes.

**Conclusions:**

The early course of PTSD symptoms is characterized by distinct and diverging response patterns that are centrally relevant to understanding the disorder and preventing its occurrence. Studies of the pathogenesis of PTSD may benefit from using clustered symptom trajectories as their dependent variables.

## Introduction

Recent events repeatedly show the extent of devastation and trauma caused by war, violence and disasters. Post-traumatic stress disorder (PTSD) transforms survivors' initial reactions to life-long illness. Chronic PTSD is prevalent, debilitating, and tenacious [1]–[3]. It occurs in a significant proportion of those who express acute PTSD symptoms after trauma exposure [4]–[6]. Preventing PTSD is a major humanitarian and public health challenge [7].

Numerous studies have shown that early, trauma-focused, cognitive behavioral therapy (CBT) reduces the prevalence of chronic PTSD among survivors with acute PTSD (e.g., [8]–[10]). However, the effectiveness of this family of resource-demanding interventions is limited by barriers to receiving care [11]–[13], by our inability to identify survivors who might remit without treatment (up to 45% of those with Acute PTSD [3], [8], [9]) as well as those who do not recover despite properly dispensed treatment (about 20%; [9]).

Previous studies of early PTSD [4], [5], [14], [15] used *central tendency statistics* to document the progressively decreasing *prevalence* of PTSD and PTSD symptoms in cohorts of survivors followed longitudinally. Subsequent meta-analyses of risk factors for PTSD [16], [17] are based on that approach. Central tendency statistics assess groups as a whole by examining change to their arithmetic mean over time. Their use implies that the mean (and dispersion around the mean) accurately and parsimoniously describes the sample studied and its reference population.

When multiple latent sub-populations are present, however, the progression of the mean does not provide an accurate picture [18], [19], in which case exploring heterogeneities of symptoms' progression better discerns underlying ‘longitudinal’ phenotypes. Uncovering these phenotypes may improve our understanding of the pathogenesis of PTSD and its early prevention.

Latent Growth Mixture Modeling (LGMM) uses maximum-likelihood estimation to uncover discrete longitudinal mixture distributions and identify latent subpopulations, or classes. Predictors of those classes, as well as the rates of change over time, can be modeled within the same framework. Studies using LGMM-based techniques to model latent subpopulations by their symptom severity have identified common patterns of response to potentially traumatic events (PTEs) and predictors of these patterns [20]–[24]. They, thereby, uncovered diagnostically meaningful patterns of stress response [18], [20], [25]. Indeed, LGMM-based techniques are emerging as a methodology to study treatment effects across disorders and identify distinct trajectories of remission, placebo response, and response to active treatment [26]–[28]. To date, however, no studies have modeled PTSD symptom progression at multiple intervals across the first year that follow trauma exposure or examined the effects of treatment in this context. This study examines *the* critical period in the formation of PTSD, namely the aftermath of trauma exposure and the effect of preventive early intervention.

The current investigation used LGMM to examine patterns of PTSD symptom progression during the fifteen months that follow traumatic events in a large cohort of trauma-exposed, initially symptomatic individuals. In an attempt to pursue the effect of treatment, we included members of this cohort who received cognitive behavioral therapy (CBT) and then examined them separately. We used LGMM's *unconditional model* to uncover clusters of symptoms trajectories in the entire cohort and LGMM *conditional model* to evaluate the effect of CBT on these trajectories.

## Methods

### Participants and Procedures

This study utilized data collected for the Jerusalem Trauma Outreach and Prevention Study (J-TOPS; [9], [13], ClinicalTrial.Gov identifier: NCT0014690) between 2004 and 2009. The J-TOPS combined a large systematic outreach and follow-up study of recent trauma survivors with an embedded, randomized, controlled trial of early interventions for survivors with acute PTSD. The study's procedures and results have been fully described in previous publications [9], [13]. The study's data is available upon request to the primary investigator (AYS). They are briefly reviewed here.

#### Screening, assessment and treatment allocation

J-TOPS's participants were adults (age: 18–70) consecutively admitted to Hadassah University Hospital emergency department (ED) following potentially traumatic events (PTEs; for full eligibility see [9], [13]). Eligible participants (n = 4,743) were screened by short telephone interviews, and those with PTEs that met DSM-IV PTSD criterion A (“a traumatic event;” n = 1996) received a structured, telephone-based interview that included an assessment of PTSD symptoms (see below). Participants with Acute PTSD symptoms in that assessment (n = 1502) were invited for clinical interviews, which only n = 756 attended. Participants with clinical-interview based Acute PTSD (save the one month duration) in the clinical assessments (n = 397) were invited for treatment unless they had chronic PTSD at the time of the traumatic event, suffered current or lifetime psychosis or bipolar disorder, or had current substance abuse or suicidal ideation. Participants who accepted the invitation (n = 296) were randomized to Prolonged Exposure therapy (PE [29]–[31]), Cognitive Therapy (CT [32]), a double-blinded SSRI/placebo condition, and a waiting list that was followed by Delayed PE at five months (for full description see [9], [13]). The results of the original study showed significant and similar efficacy for all three CBT-based interventions (PE, Late-PE, and CT). In this work, we collectively refer to these interventions as ‘CBT.’ The effect of the SSRI (escitalopram) did not differ from placebo or waitlist.

#### Follow-up

Unrelated to treatment eligibility or participation, the J-TOP included a large follow-up study. Participants seen at 10 days (n = 1996) were re-evaluated *seven* (n = 1784) and *fifteen* (n = 1022) months after ED admission. Participants of the first clinical assessment (n = 756) were re-evaluated *five months* after the traumatic event (n = 604) regardless of treatment participation. Telephone- and clinical interviewers were blind to subjects' participation in the embedded steps (i.e., attending clinical interviews for telephone interviewers and attending treatment for clinical interviewers). Participants provided oral consent to be interviewed by telephone and written informed consent for clinical assessments, randomization, and treatment. All procedures were approved and monitored by the Hadassah University Hospital's institutional review board.

#### Current Study Sample

We utilized individuals who had data available at ten days and at least two additional time points. Additionally, individuals whose data were collected at inconsistent time intervals from the rest of the sample (as determined by being further than two standard deviations from the mean data collection time for each assessment) were not included. The final sample for the current study was n = 957, with 125 receiving CBT (PE: n = 49; CT = 41; Late PE n = 35). The mean age of the current sample was 36.29 years (SD = 12.04). Mean length of stay in the emergency room was 5.72 hours (SD = 6.31). Individuals in the current sample came to the emergency room primarily due to motor vehicle accidents (84.1%) followed by terrorist attacks (9.4%), then work accidents (4.4%) then other types of incidents (2.0%).

We assessed if individuals who were included in this work differed from those excluded from the analysis. Using a Pearson's χ^2^, we compared those who were retained from those who were removed on gender [χ^2^ (1,1501) = .08, *p* = .78], and on reported exposure to a PTE (with three levels indicating no exposure, exposure to the same type of event, and exposure to another type of event [χ^2^ (2,1500) = 3.80, *p* = .15]). Using an independent samples t-test, we also compared those who were included with those excluded on age [t (2, 1500) = −0.55, *p* = .59], general distress at 10 days (see instruments below; [t (2, 1500) = −1.04, *p* = .30], and PTSD symptoms at 10 days [t (2, 1500) = −1.78, *p* = .08]. We further examined the trend difference in initial PTSD symptoms score and found that that these groups were substantively non-distinct (respectively, for those included and excluded, mean PSS-I scores were 10.70, SD = 3.11 vs. 10.41, SD = 3.10). We also conducted an analysis of variance (ANOVA) to estimate effect size of the difference and found a trivial effect (η^2^ = .002). Finally, we compared the demographics and initial PTSD symptom severity of those who were removed because they fell more than two standard deviations outside of the mean data collection dates (n = 40). These individuals did not significantly differ in age [t (2, 995) = 1.15, *p* = .25], initial symptom levels at the first interview [t (2, 995) = −1.64, *p* = .10], or gender [χ^2^(1, 996) = 0.21, *p* = .65]. As such, we concluded that individuals who were removed to improve the analysis were not a substantively distinct population from those who were retained.

#### Timing of assessments

Successive telephone assessments in this sample took place, respectively 9.21 SD = 3.20, 221.34 SD = 33.90 and 468.07 SD = 109.32 days after ED admission. We refer to these time lags as ‘ten days,’ ‘seven months’ and ‘fifteen months.’ The clinical interviews took place 29.51 SD = 4.93 and 143.00 SD = 32.33 days after ED admission (alias ‘one month’ and ‘five months’).

### Instruments

*The Clinician-Administered PTSD Scale* (CAPS) [33] is a widely used structured clinical interview for evaluating the presence of PTSD and the severity of PTSD symptoms. In this study, the CAPS was administered during clinical assessments only, and thus was not useful as a measure of symptom trajectories. We use it to evaluate the concurrent validity of the PTSD Symptom Scale (below).

*Structured Clinical Interview for DSM-IV* (SCID) [34] is a widely used structured clinical interview for evaluating the presence of DSM-IV symptoms and diagnostic status. In this study, the SCID was administered during the clinical assessments only. We utilized this scale to examine the prevalence of anxiety disorders and Major Depressive Disorder current and lifetime diagnoses broadly in this sample and as they relate to individuals who fall into the modeled trajectories. Because the entire sample did not receive a clinical interview, however, this data is only presented on the subset that did.

*The PTSD Symptom Scale (PSS)* quantified PTSD symptoms at all time-points. The PSS is a structured, diagnostic instrument that follows DSM-IV 17 PTSD symptom criteria [35]. The PSS interviewer version (PSS-I; [35]) was used during telephone interviews, with items dichotomized into present vs. absent statements about each PTSD symptom criterion (score range: 1 to 17). The self-administered version of the PSS (PSS-SR; [36]) was used during clinical assessments. This version uses a 1–4 symptom severity score for each item. A score of two or more was considered an endorsement of the presence of a symptom (score range: 1 to 17). The PSS-SR total scores during the *clinical interviews* were highly correlated with concurrent CAPS total scores (at one month: *r* = .77, *p*<.001; at five months: *r* = .84, *p*<.001). Measurement equivalence between telephone-based PSS-I and clinically administered PSS-SR scales was established by examining the correlations between the proximal five months clinical interviews and seven months telephone interviews. The Pearson's correlation coefficient revealed a strong relationship between the scores (*r* = .75, *p*<.001). Additionally, telephone-based PSS-I scores at seven months correlated significantly with the five months *CAPS* total score (*r* = .76, *p*<.001). Based on this evidence of measurement equivalence, we conducted our analysis utilizing both PSS-I dichotomous scores and in-person PSS-R dichotomized symptom scores.

*The Kessler-6 (K6)* is a brief 6-item self-report instrument that measures general distress. It was administered during telephone interviews. The K6 items are rated on a five-point scale from zero (“none of the time”) to four (“all of the time”), yielding a total score ranging from 0 to 24. The K6 strongly discriminates between community cases and non-cases of DSM-IV/SCID disorders with Receiver Operating Characteristic (ROC) curve of 0.87–0.88 for all disorders with Global Assessment of Functioning (GAF) scores of 0–70 and 0.95–0.96 when disorders had GAF scores of 0–50 [37].

*The occurrence of new PTEs during follow-up* was evaluated by asking subjects, during seven and fifteen months' interviews, whether they incurred a traumatic event since their inclusion in the study. Responses were coded as ‘no incident’, ‘incident of the same nature’ and ‘different incident.’ This variable was dummy coded for trajectory analysis to indicate presence/absence of any recent incident.

### Data Analytic Plan

We utilized Mplus 6.0 [38], employing robust full information maximum-likelihood (FIML) procedures to identify heterogeneous latent classes of PTSD symptom severity over time using LGMM. These modeling techniques allowed us to test whether the population under study is composed of a mixture of discrete distributions characterized as classes of individuals with differing profiles of growth [39], while also allowing for the modeling of covariates as predictors of class membership and slope parameters [40].

#### Unconditional Model

We compared a progressive number of classes characterized by linear only or linear and quadratic parameters while accounting for non-specific psychological distress by residualizing PTSD symptom scores at 10 days and 7 months on K6 scores. We accounted for the effect of eventual *trauma exposure during the study* by regressing PTSD symptom scores at 7 and 15 months on our dummy-coded trauma-re-exposure variable as a time variant covariate. We compared progressive nested trajectory models by assessing relative fit based on reductions in the Bayesian Information Criterion (BIC), sample-size adjusted Bayesian Information Criterion (SSBIC), Aikaike Information Criterion (AIC), and significance indicated by the Bootstrap Likelihood Ratio Test (BLRT), along with parsimony and interpretability equally weighed. Entropy was also examined but not utilized to determine the number of classes; all criteria were consistent with recommendations from the literature [41].

#### Conditional Model

After establishing our best-fitting model, we first regressed class membership and then the freely estimated slopes within each class on a dummy-coded variable indicating the receipt of treatment. Next, we examined further covariates as predictors of the classes including *age, gender*, and ten days symptom severity in the three clusters of PTSD symptomatology including *avoidance, arousal*, and *intrusions*. We analyzed these separately from the treatment variable because we wanted to examine the effect of the treatment variable.

## Results

### Symptom progression in the entire sample

By examining mean level PTSD symptom severity across our five measurement points, we found that, as mean level symptoms decrease in the entire sample, the standard deviation increases, indicating that the mean is characterizing an increasingly wide distribution of symptoms and suggesting that the distribution is becoming increasingly non-normal (**Table 1**).

**Table 1:** Study Groups' Comparisons.

	Non-Remitting (1)		Slow Remitting (2)		Rapid Remitting (3)		Total Sample		F (df)/X^2^	^Post-hoc^
	*M* (SD) n(%)	95% CI	*M* (SD) n(%)	95% CI	*M* (SD) n(%)	95% CI	*M* (SD) n(%)	95% CI		
	(n = 163)		(n = 258)		n = 536		(n = 957)			
**Gender (% Male)**	74(45%)		137 (53%)		280(52%)		491(51%)		2.80(2,954) = .25	1 = 2 = 3
**Received CBT**	26 (19%)		38 (17%)		61 (13%)		125		1.89(2,954) = .39	1 = 2 = 3
**Ten Days**	**(n = 163)**		**(n = 258)**		**(n = 536)**		**(n = 957)**			
**PTSD Symptoms**	12.17(3.14)	11.68, 12.65	10.39(3.16)	10.00, 10.78	9.71 (3.09)	9.44, 9.97	10.31(3.24)	10.10,10.51	38.98(2,954)<.001	1>2>3
**One Month**	**(n = 93)**		**(n = 135)**		**(n = 286)**		**(n = 514)**			
**PTSD symptoms**	14.87(2.31)	14.38, 15.35	13.50(2.95)	13.00, 14.00	10.14 (4.22)	9.65,10.64	11.87(4.13)	10.10,10.51	80.08(2,511)<.001	1>2>3
PTSD^*^ (%)	97 (96%)		128 (91%)		186 (61%)		401 (73%)		72.53(2,511)<.001	1,2>3
**Five Months**	**(n = 88)**		**(n = 115)**		**(n = 260)**		**(n = 463)**			
**PTSD symptoms**	14.59(2.26)	14.09, 15.08	10.15(3.85)	9.43, 10.87	3.54 (3.00)	3.17, 3.92	7.27(5.44)	6.76, 7.78	404.52(2,460)<.001	1>2>3
**PTSD (%)**	93 (98%)		82 (65%)		21 (8%)		196(40%)		280.14(2,460)<.001	1>2>3
**Seven Months**	**(n = 163)**		**(n = 255)**		**(n = 533)**		**(n = 951)**			
**PTSD symptoms**	13.01(2.71)	12.59, 13.43	7.95(2.77)	7.61, 8.29	2.67 (2.08)	2.50, 2.85	5.86(4.62)	5.57, 6.16	1297.17(2,948)<.001	1>2>3
**PTSD (%)**	151 (93%)		124(49%)		16 (3%)		291(31%)		525.60(2,948)<.001	1>2>3
**15 Months**	**(n = 141)**		**(n = 225)**		**(n = 472)**		**(n = 838)**			
**PTSD symptoms**	12.17(2.37)	11.78, 12.56	6.19(2.87)	5.81, 6.57	1.78 (1.80)	1.61, 1.94	4.71(4.45)	4.41, 5.01	1248.97(2,835)<.001	1>2>3
**PTSD (%)**	129 (91%)		57(25%)		6 (1%)		192 (23%)		501.34(2,835)<.001	1>2>3
**PSS scores among PTSD participants**	12.48 (2.18)		9.54 (1.78)		8.17 (0.98)				204.35(2,189)<.001	1,2>3

### Treatment allocation

We examined differences between those who received CBT and those who did not. No significant difference was observed between the treatment and non-treatment groups by gender [χ^2^ (1, 956) = 2.44, *p* = .12)]. Significant differences between these groups were observed on *age*, the treatment group being slightly older (respectively, in years, 39.30, SD = 12.25 vs. 35.77, SD = 11.79; *t* (1, 956) = −3.11, *p*<.01). Participants who received treatment had higher *PTSD symptom severity at 10 days (i.e.*, prior to treatment initiation) (PSS-I total score = 11.63, SD = 2.85 vs. 10.11, SD = 3.25; *t* (1, 956) = −4.95, *p*<.001) and higher *ten days' K6 scores* prior to treatment initiation (mean = 19.15, SD = 4.45 vs. 17.60, SD = 5.26; *t* (1, 956) = −3.14, *p*<.01). Because t-tests are sensitive to sample size, we examined the effect size by group, using a one-way ANOVA and those were as follows: for *age* (η^2^ = .01), for *ten days' PSS-I scores* (η^2^ = .03) and for *10 days K6* (η^2^ = .01). Based on accepted psychometric standards [42], we concluded that differences between groups were trivial.

### Unconditional Model: uncovering latent classes

Based on the AIC, BIC, SSBIC, and BLRT, we found that successive models continued to demonstrate improved fit through four classes, both with linear only, and linear+quadratic parameters, with linear+quadratic parameters consistently out-performing linear alone (**Table 2**). However, both with linear only and linear+quadratic parameters, the addition of a fourth class served only to split a class into two parallel trajectories with no substantive distinction in symptom levels. As a result, the four-class model was rejected for being less parsimonious and less interpretable, and *the three-class model with linear+quadratic parameters was retained*.

**Table 2:** Fit Indices for One- to Four-Class Growth Mixture Models of PTSD Symptom Severity (n = 957).

	Linear Weights Only				Linear+Quadratic Weights			
Fit Indices	1 Class	2 Classes	3 Classes	4 Classes	1 Class	2 Classes	3 Classes	4 Classes
AIC	31356.07	31129.22	31062.93	30980.84	30994.95	30559.06	30466.79	30388.28
BIC	31448.48	31245.95	31203.98	31146.21	31092.23	30658.52	30622.43	30573.11
SSBIC	31388.15	31169.73	3111.88	31038.23	31028.71	30602.94	30520.80	30452.42
Entropy	—	.84	.82	.82	—	.84	.78	.79
BLRT	—	*p*<.001	*p*<.001	*p*<.001	—	*p*<.001	*p*<.001	*p*<.001

*Note.* AIC = Akaike information criterion; BIC = Bayesian information criterion; SSBIC = sample size adjusted Bayesian information criterion; LRT = Lo-Mendell-Rubin test; BLRT = bootstrap likelihood ratio test.

**Figure 1:**
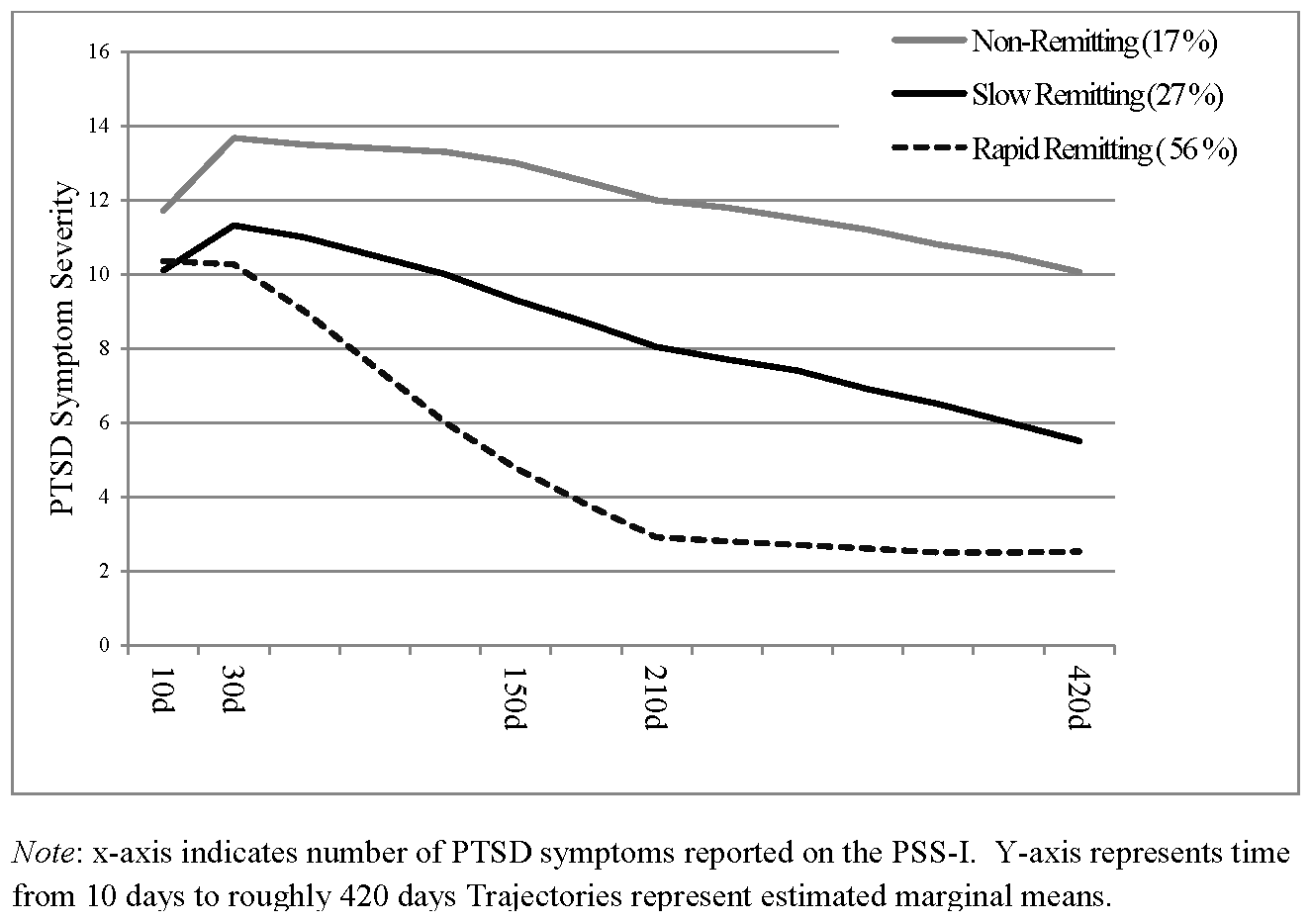
Three Trajectory Model of PTSD Symptom Severity Recovery Trajectories (n = 957).

This model identified three substantively distinct classes. The largest class (**Rapid Remitting; 56% of the sample**) displayed a precipitous drop in symptoms from 1 to 5 months as captured by a significant negative slope (*Est* = −26.72, *SE* = 2.28, *p*<.001), indicating a significant overall drop in symptoms from 10 days to five months, accompanied by a significant positive quadratic parameter, indicating a curvilinear rate of change (*Est* = 17.16.23, *SE* = 1.93, *p*<.001). The second largest class (**Slow Remitting; 27%**) demonstrated a relatively consistent rate of symptom reduction across time points, as indicated by a significant negative slope (*Est* = −8.83, *SE* = 2.50, *p*<.001) and a non-significant quadratic parameter (*Est* = 1.95, *SE* = 0.63, *p* = .23). Finally, the smallest class (**Non-Remitting; 17%**) demonstrated consistently high symptom severity across time points with no significant change over time, indicated by a non-significant slope (*Est* = −1.19, *SE* = 1.68, *p* = .48) and a non-significant quadratic parameter (*Est* = −2.67, *SE* = 1.62, *p* = .10; **Figure 1**). Members of the rapid remitting class also reached lower PTSD symptom levels at 15 months compared to those of slow remitting class, and the latter had lower levels of PTSD symptoms than the non-remitting class (Table 1). The frequency of full PTSD in the entire sample is 21.8% while rates of sub-syndromal PTSD based on meeting at least 2 of the three symptom cluster criteria is 15.8% based on the PSS.

To assess trajectories while accounting for general levels of distress, we regressed symptom levels at 10 days and 7 months on initial K6 scores. These variables improved entropy indicating that accounting for general distress improves identification of class membership. Levels of general distress at 10 days were significantly positively associated with PTSD symptom at 10 days (*Est* = 0.11, *SE* = 0.01, *p*<.001), and marginally so at 7 months *(Est* = 0.02, *SE* = 0.01, *p* = .07) across the entire population.

Novel trauma exposure, during the study, was not significantly associated with differences between classes in concurrent PTSD symptom levels at seven (*Est* = 0.07, *SE* = 0.24, *p* = .76) and 15 months (*Est* = 0.34, *SE* = 0.20, *p* = .08).

Finally, to assess if the trajectories were biased by the selection of individuals with 3 or more time points, we conducted the same analysis with all the participants. This analysis revealed weaker overall fit in terms of entropy, but recovered the same classes in roughly the same proportions.

### Conditional model: effect of treatment and other covariates on latent trajectory classes

To examine the effect of treatment on the LGMM parameters we first we regressed class membership on our dummy-coded yes/no treatment variable, conducted in the MPlus environment using a multinomial logistic regression. Results of these analyses did not approach significance suggesting that receiving treatment did not affect class membership.

Following the examination of the effects of treatment on class, we explored further covariates as predictors of the latent classes using the same modeling framework. We examined the following variables: *gender, age*, and total levels of PTSD symptomatology at 10 days based on the three symptom domains (*intrusions, avoidance, arousal*). Gender and intrusions were not significantly different between the three identified classes and none of these covariates differentiated the Rapid and the Slow Remitting classes. Compared to the Rapid Remitting class, however, the Non-Remitting class was significantly older (*Est* = 0.04, *SE* = 0.01, *p*<.001), marginally more likely to have higher levels of avoidance symptomatology (*Est* = 0.11, *SE* = 0.06, *p* = .10) and significantly more likely to have higher levels of arousal symptomatology (*Est* = 0.26, *SE* = 0.09, *p*<.01). This class was also significantly more likely to be older then the Slow Remitting class (*Est* = 0.02, *SE* = 0.01, *p*<.05) and more likely to have significantly higher levels of both avoidance (*Est* = 0.19, *SE* = 0.08, *p*<.05) and arousal symptom severity (*Est* = 0.22, *SE* = 0.10, *p*<.05).

Next, we regressed the random slope parameter of each class on the treatment variable while controlling for distress at 10 days. General distress at 10 days significantly predicted the slopes *across classes* (*Est* = −0.42, *SE* = 0.04, *p*<.001). However, this analysis revealed non-significant effect of treatment on the Rapid Remitting and the Non-Remitting Classes and a significant negative effect in the Slow Remitting Class (**Table 3**). These findings indicate that individuals in the Slow Remitting Class, but not other classes, benefit from treatment: treatment serves to accelerate their symptom decline over time.

**Table 3:** Growth Factor Parameter Estimates for Treatment on the Slope of the 3-Classes (n = 957).

Class	*Est.*	*S.E.*	*p< = *
Slow Remitting	−0.96	0.49	0.05
Rapid Remitting	1.52	1.28	0.23
Non-Remitting	1.33	1.05	0.20

*Note*. *Est = Estimate; SE = Standard Error.*

In the above analyses we retained individuals who received late PE because of concerns that removing them from the analyses could bias the sample. Hypothetically, however early and delayed PE could have differentially affected symptom trajectories. We therefore repeated the analysis with these individuals removed, which resulted in similar effect of treatment on class membership and slopes.

### Post-hoc analyses: Trajectories, PTSD, other Diagnoses, and Demographics

We examined the relationship between the LGMM-identified trajectories and *meeting PTSD diagnostic criteria* at different time points. We also examined *gender differences* by class. To conduct this analysis we saved the most probable class assignments for analysis outside of the model and conducted a series of χ^2^ comparisons in SPSS 19. The classes differed in the *likelihood of meeting PTSD diagnostic criteria* at five, seven and fifteen months (Table 1). There were no significant differences by gender in relation to class, and no statistically significant differences the proportion of *individuals in treatment* by class (Table 1). We also examined differences in age between the classes using a two-tailed ANOVA. The overall test was significant [*F* (2,954) = 11.60, *p*<.001]; however, the effect size was trivial (η^2^ = .02).

Next by comparing mean symptoms and the standard deviation from the mean, we observe no noticeable reduction in PTSD symptom levels in the Non-Remitting class from 10 days (μ = 12.17, SD = 3.14) to 15 months (μ = 12.17, SD = 2.37), a moderate reduction in total symptoms in the Slow Remitting class from 10 days (μ = 10.39, SD = 3.16) to 15 months (μ = 6.19, SD = 2.87), and a large reduction in total symptoms in the Rapid Remitting class from 10 days (μ = 9.71, SD = 3.09) to 15 months (μ = 1.78, SD = 1.80). The resulting confidence intervals indicate separation between classes at all time-points (Table 1).

Finally, we examined the prevalence of one month DSM IV Anxiety Disorders (i.e., any anxiety disorder other than PTSD) and Major Depressive Disorder (MDD) among participants who attended the first clinical interview (n = 514) and conducted a series of pearson χ^2^ analyses to test if meeting these diagnoses differed between latent trajectory classes. The prevalence of anxiety disorders in the entire sample was 27.8% (n = 143) and that of current MDD 38.5% (n = 198). The trajectory groups had similar prevalence of current anxiety disorders. They differed, however in the prevalence of current MDD [(respectively for Non Remitting, Slow Remitting and Rapid Remitting 66.0% 47.4% and 21.2%; χ^2^(4, 423) = 76.58, *p*<.001] with significant differences between every two trajectory groups (for Slow Remitting vs. Rapid Remitting [χ^2^(1, 426) = 31.65, *p*<.001]; for Non-Remitting vs. Slow Remitting [χ^2^(1, 225) = 8.23, *p*<.01] for Non-Remitting vs. the Rapid Remitting [χ^2^(1, 374) = 70.25, *p*<.001]).

## Discussion

The current study evaluated the occurrence of latent classes characterized by their trajectory of symptom change from 10 days to 15 months post-trauma among a large cohort of recent trauma survivors. Among 957 who were followed 125 (13.1%) received efficacious CBT and we tested the relationship between receiving treatment and the identified trajectories.

We identified three latent classes of symptom change: A large class characterized by a precipitous drop in symptoms from one to five month (Rapid Remitting, 56%), a class characterized by a slow linear decline of symptoms over 15 months (Slow Remitting, 27%) and a class characterized by a failure to remit and no reduction in symptoms (Non-remitting, 17%).

We also examined demographic and symptom levels at 10-days as predictors of symptom trajectory classes and found that the Non-remitting class was predictable by older age, higher levels of initial hyperarousal symptoms and, less consistently, elevated avoidance symptoms. Testing the robustness of these and other putative predictors requires in-depth classifier analyses of this and other longitudinal.

Examining the relationship between receipt of treatment and the three classes we, firstly, found no evidence that receiving treatment affected class membership and secondly found that, within classes, treatment accelerated the rate of recovery in the *Slow Remitting* class alone and had no effect on the two other classes.

As such, these findings indicate the early CBT is effective – or necessary - for a subset of symptomatic trauma survivors. The finding concerning unnecessary CBT for rapid remitters replicates a previous finding of our group [9] and other groups [43]. However, *the occurrence of a non-remitting and treatment resistant group is a novelty*. Importantly, in both non-remitting and rapid remitting groups, treatment was followed by an apparent improvement, but such improvement did not differ from the spontaneous recovery of those untreated within each group. The relatively small proportion of subjects in the non-remitting group emphasizes the contribution of the latent trajectory approach to discerning pertinent outcome groups within entire cohorts. These findings have broad relevance for understand the natural course of PTSD, the differential effects of treatment, and the heuristics of further discovery.

*Regarding the Natural Course of PTSD*, our findings indicate that heterogeneities in individuals' symptom trajectories following trauma are not random events, but rather cluster into typical, minimally overlapping subsets. Our findings also suggest that the resulting subsets are highly informative with regard to the occurrence and the severity of chronic PTSD. These populations appear to be more informative and less error prone then the use of diagnostic status as an outcome. Firstly, we find the 91% of individuals who qualify for a PTSD diagnosis at 15-months fall into the *Non-Remitting* trajectory. Further, among those who meet PTSD criteria at 15 months those in the non remitting group have significantly higher symptom severity Differences in symptom severity at fifteen months suggest that individuals on the slow and rapid remitting group who still meet PTSD symptom criteria might be on their way to recovery.

Our work differs from previously reported studies (i.e. [22], [44]) in that it does not include survivors without initial significant elevations in symptoms. As a previous analysis of these data has shown [9], [14], such individuals are very unlikely to develop PTSD. The current results reflect, therefore, symptom trajectories among survivors at high risk – rather than among entire cohorts of individuals exposed to potentially-traumatic events. In the context of the current study, we strove to identify heterogeneous responses among those who are initially highly symptomatic, to attempt to predict these sub-populations, and to examine the differential effects of treatment as it relates to these sub-populations.

From a *treatment and prevention* perspective, the finding of an *unremitting and treatment-resistant* trajectory is equally important. First, the majority of patients with chronic PTSD at 15 months (n = 129 of n = 192; 67.2%) come from this small group. Second, symptom levels of those who remain with 15 months' PTSD in the non-remitting group are significantly and meaningfully higher than those of the other classes (30.1% higher than in the slow remitting group and 52.7% higher than the rapid remitting group), evoking the question of fundamental differences between the resulting conditions (e.g., potential for further recovery, neuro-cognitive underpinning). It is therefore important to further explore this group, in this and subsequent studies.

Looking at ways to predict this group, the non-remitting group in this work separated from the other groups as early as 10 days after the traumatic event (symptom levels and confidence intervals do not overlap). However, this post-hoc observation is not yet mature for clinical use as a predictor nor is it informative about underlying neuro-behavioral mechanisms. Attaching biographical information (e.g., prior trauma, childhood adversity) as well as neuropsychological, biological and recovery-environment factors to this trajectory may lead to better – and specific - understanding of this catastrophic course of early PTSD symptoms.

The non-remitting group should also be amenable, as such, to longitudinal neuro-cognitive and neuro-imaging studies looking into putative changes in the ways the CNS transmutes an initial reaction into chronic, entrenched disturbance. Finding analogous trajectories in PTSD-related biomarkers would buttress this ‘irreversible acquisition’ trajectory in biological findings. Recent and similar trajectories in animal models of conditioned fear provide encouraging evidence to the existence of such analogies [45], [46]. Better understanding the dynamics of non-remission may hold a key for further discovery other mental disorders with identifiable onset and non-remitting course in a subset of patients.

Our unexpected finding of treatment (CBT) resistance in this group makes these patients eager candidates for other treatment approaches. However, *even when effective*, novel therapies for small proportion of survivors are unlikely to generate a significant signal in studies of entire affected groups. This highlights the importance of identifying pertinent subpopulations for assessing treatment effects: one treatment could be highly effective in the aggregate while ineffective for a minority – and vice versa. Indeed, the use of LGMM has already revealed informative description of distinct courses of recovery in randomized clinical trials of depression, in which it differentiated the effects of treatment from that of natural recovery and placebo [27], [28], [47]. These efforts are in line with the emergence of trait-sanctioned therapies for medical conditions (e.g., receptor-specific therapies for breast cancer, multiple myeloma).

The *slow remitting* trajectory is similarly interesting. The unique effect of treatment on members of this cluster suggests a special sensitivity to the effects of CBT, and thus might allow a better allocation of patients to early treatment. It would be interesting as well to explore the reasons for such responsiveness via exploring membership in this trajectory class.

The finding of positive treatment effect in this otherwise progressively remitting class is also in line with a previous and very intriguing observation from epidemiological studies [3], according to which early treatment (though studied retrospectively) accelerated recovery but did not reduce the overall burden of PTSD. Granted, accelerating recovery by months or years has profound clinical and personal implications. Nonetheless, the putative category of ‘recover-able’ trauma survivors is extremely interesting to follow as it may optimally teach us about recovery mechanisms that may not exist in the other two groups, and how to engage them. Again – studying recovery in entire cohorts may not be sensitive enough.

The finding of a *rapidly remitting subgroup* is in line with previous CBT studies, in which patients with less than full Acute PTSD symptoms recovered with or without treatment [8], [9]. Identifying who will follow this course has broad public health implications, as it could lead to the better allocating survivors to therapy and better use of treatment resources.

Further, our observation can inform the *heuristics of uncovering the pathogenesis of PTSD*. The finding of pertinent classes of symptom trajectories challenges the use of central tendency statistics to enhance discovery in the area of nascent PTSD. Central tendency statistics may collapse heterogeneous populations and obfuscate the identification of relevant subpopulations. To take advantage of the methodology presented here, future studies should collect multiple data points at timing and intervals that are critical for understanding the underlying problems, and with an eye towards imputation of missing cases (e.g., by collecting enriched initial assessments).

We found some indication that current depression differentiates trajectories. These data are limited, however, because full clinical assessments were not conducted on the entire cohort. This is potentially valuable information as it indicates that depression symptomatology in the acute phase may be predictive of chronic posttraumatic stress. This finding is consistent with other findings in the literature that have demonstrated that depression, in part, influences the development and maintenance of PTSD [48]. Finally, despite evaluating the same construct (PTSD symptoms) and establishing measurement equivalence, the use of different versions of the PSS at different time points should be seen as limitation of this study.

## Conclusion

This work uncovered one of, possibly, several symptom trajectory scenarios in recent trauma survivors. Rape survivors, or victims of repeated or protracted violence, may have different longitudinal paths. This may also be true in deployed combatants, whose survival in a battlefield may require a suppression of initial symptoms, and result in their delayed emergence [49]. Nonetheless the approach outlined here emphasized a robust methodology for uncovering systematic clustering patterns within response heterogeneities. Its ultimate challenge will be its ability to better inform clinical and biological studies of the pathogenesis of trauma and stress-related disorders and uncover robust predictors of symptoms persistence and chronicity.
