# Correction: Early PTSD Symptom Trajectories: Persistence, Recovery, and Response to Treatment: Results from the Jerusalem Trauma Outreach and Prevention Study (J-TOPS)

**DOI:** 10.1371/annotation/0af0b6c6-ac23-4fe9-a692-f5c30a3a30b3

**Published:** 2013-08-27

**Authors:** Isaac R. Galatzer-Levy, Yael Ankri, Sara Freedman, Yossi Israeli-Shalev, Pablo Roitman, Moran Gilad, Arieh Y. Shalev

There was information omitted from the Funding statement. The correct version of the Funding statement is available below:

This work was supported by a service development grant from the Jewish Federation of New York and a research grant MH071651 from the National Institute of Mental Health. Dr. YIS received an investigator-initiated grant from Lundbeck Pharmaceuticals for this study and for a collaborative study (principal investigator: Dr. Joseph Zohar) entitled “Prevention of PTSD by Escitalopram.” The funders had no role in study design, data collection and analysis, decision to publish, or preparation of the manuscript. This does not alter the authors' adherence to all the PLOS ONE policies on sharing data and materials. 

